# Is Vestibular Neuritis an Immune Related Vestibular Neuropathy Inducing Vertigo?

**DOI:** 10.1155/2014/459048

**Published:** 2014-01-15

**Authors:** A. Greco, G. F. Macri, A. Gallo, M. Fusconi, A. De Virgilio, G. Pagliuca, C. Marinelli, M. de Vincentiis

**Affiliations:** Organs of Sense Department, ENT Section, Policlinico “Umberto I” University of Rome “Sapienza”, Lgo Valerio Bacigalupo 32 C, 00142 Rome, Italy

## Abstract

*Objectives*. To review the current knowledge of the aetiology of vestibular neuritis including viral infections, vascular occlusion, and immunomediated mechanisms and to discuss the pathogenesis with relevance to pharmacotherapy. *Systematic Review Methodology*. Relevant publications on the aetiology and treatment of vestibular neuritis from 1909 to 2013 were analysed. *Results and Conclusions*. Vestibular neuritis is the second most common cause of peripheral vestibular vertigo and is due to a sudden unilateral loss of vestibular function. Vestibular neuronitis is a disorder thought to represent the vestibular-nerve equivalent of sudden sensorineural hearing loss. Histopathological studies of patients who died from unrelated clinical problems have demonstrated degeneration of the superior vestibular nerve. The characteristic signs and symptoms include sudden and prolonged vertigo, the absence of auditory symptoms, and the absence of other neurological symptoms. The aetiology and pathogenesis of the condition remain unknown. Proposed theories of causation include viral infections, vascular occlusion, and immunomediated mechanisms. The management of vestibular neuritis involves symptomatic treatment with antivertiginous drugs, causal treatment with corticosteroids, and physical therapy. Antiviral agents did not improve the outcomes.

## 1. Introduction

Vestibular neuritis (VN) is the second most common cause of peripheral vestibular vertigo (the first being Benign Paroxysmal Positional Vertigo) and is a disorder thought to represent the vestibular-nerve equivalent of sudden sensorineural hearing loss.

The first case of VN was reported by Ruttin in 1909 [1], and the term was coined by Hallpike in 1949 [2] and Dix and Hallpike in 1952 [3]. Synonyms for vestibular neuritis include acute labyrinthitis [4], acute unilateral vestibular paralysis [5], and epidemic vertigo [6].

Vestibular neuronitis is caused by a sudden unilateral loss of vestibular function. In the majority of cases, the functions of the organs that are innervated by the superior vestibular nerve (the superior and lateral semicircular canals and the utricle) are damaged, but the functions of the organs that are innervated by the inferior vestibular nerve (the posterior semicircular canal and the saccule) are spared [7].

Vestibular neuritis has an incidence of approximately 3.5 cases per 100,000 people [8]. The typical age of onset is between 30 and 60 years, and the age distribution plateau is between 40 and 50 years [8]. There is no significant gender difference, and 30% of all affected patients had common colds prior to developing the disease.

In a recent long-term follow-up study of 103 patients with vestibular neuritis, only two patients (1.9%) developed a second case of vestibular neuritis, which occurred 29 to 39 months after the first vestibular neuritis case [9]. The second case affected the contralateral ear in both patients. Unlike Bell's palsy and sudden hearing loss, a relapse in the same ear does not occur.

Cases of vestibular neuronitis are typically sporadic [10], but they have been frequently observed to occur in epidemics [11]. One early account of the disease described an epidemic among farmers and their labourers in a canton in Geneva in 1888 [12], and Walford described an epidemic that occurred in an artists' colony in Chelsea, London [13]. In that epidemic, the condition was known locally as “the staggers.”

The characteristic signs and symptoms of vestibular neuritis include sudden, severe, and prolonged vertigo measured over several days, the absence of auditory symptoms (deafness or tinnitus), and the absence of other neurological symptoms (particular diplopia or dysarthria).

Vestibular neuronitis is a benign condition. The severe initial phase of the disease usually lasts between two and three days, but it may last a week or longer. The course of the entire illness averages six weeks [14], but it may last nine weeks or longer [15].

The complete or partial resolution of vestibular symptoms is standard in vestibular neuritis. In a clinical follow-up of 38 patients with vestibular neuritis, Matsuo and Sekitani [16] demonstrated that at least 15% had significant vestibular symptoms even after l year. The prognosis may be better in children [17].

Vestibular neuronitis is diagnosed using clinical diagnostic criteria, and there is no specific investigation to confirm the diagnosis. Meniere's disease can almost always be excluded on the basis of the absence of hearing loss. In rare cases, hearing loss can be absent early in the course of Meniere's disease. If the diagnosis is unclear, the character of the vertigo may help to differentiate between the two conditions. The vertigo of Meniere's disease is characteristically episodic and lasts a few hours [18], while the vertigo of vestibular neuronitis is usually constant and lasts several days.

**Figure 1:**
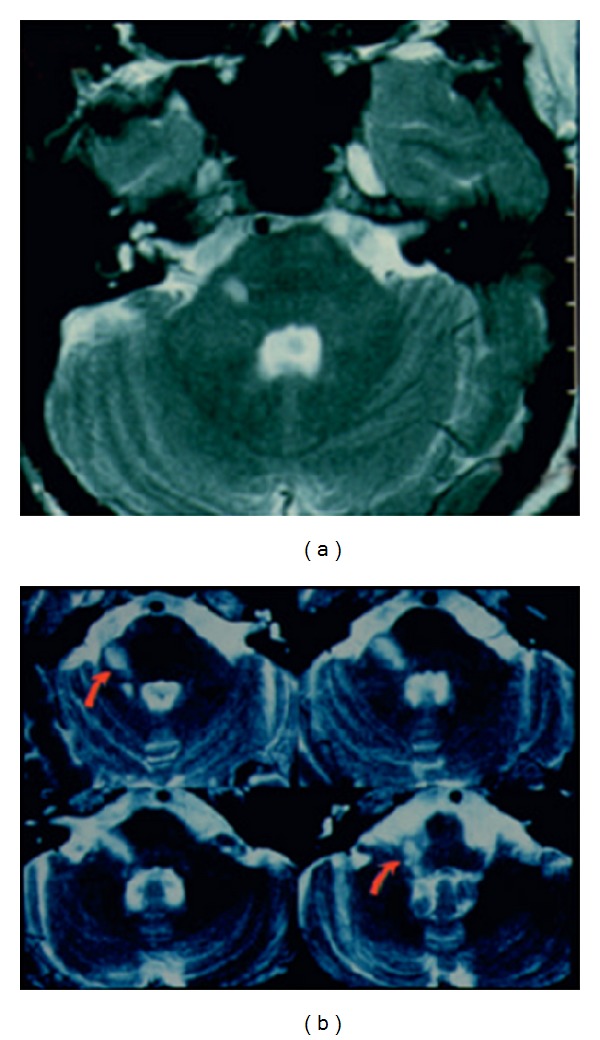
(a) (MRI-T2 image) Fascicular and nuclear lesion of the vestibular nerve due to a multiple sclerosis plaque mimicking vestibular neuritis. (b) (MRI-T2 image) Fascicular and nuclear lesion of the vestibular nerve due to a vascular lesion mimicking vestibular neuritis.

In younger adult some condition to consider in the diagnosis is multiple sclerosis. Multiple sclerosis rarely presents as isolated vertigo, and when multiple sclerosis presents with isolated vertigo, it may be impossible to distinguish it from vestibular neuronitis until further evidence of central nervous system involvement emerges. The differential diagnosis is difficult, and the final diagnosis is confirmed by neuroimaging [19] (Figure 1).

It is difficult to identify patients with “pseudoneuritis” secondary to an ischemic stroke in the posterior fossa [19], especially in the territory of the posterior inferior cerebellar artery (PICA) [20, 21]. Computed tomographic scans of the brain are not as sensitive as RMI for an acute ischemic stroke in the posterior fossa [22].

The differential diagnosis of peripheral labyrinthine and vestibular nerve disorders that mimic vestibular neuritis includes several rare conditions.

Initial burning pain and blisters that occur with hearing disorders and facial paresis are typical for herpes zoster oticus (Ramsay-Hunt syndrome).

Cogan syndrome is a severe autoimmune disease accompanied by interstitial keratitis and audiovestibular symptoms [23].

Vogt-Koyanagi-Harada syndrome is a rare multisystemic disease that affects tissues containing melanin, including the eye, inner ear, meninges, and skin [24].

Histopathological studies of patients who died after the onset of symptoms of unrelated clinical problems demonstrated isolated degeneration of the superior vestibular nerve, vestibular neuroepithelium, and vestibular ganglion [25] with a deficiency in the population of the nerve fibres [26]. Light and electron microscopic findings of myelin degeneration were also obtained [27]. One study demonstrated the associated thrombosis of a large vessel in the internal auditory meatus [28], but another study could find no evidence of vascular occlusion in the four specimens studied [26].

## 2. Pathogenesis

It is a well established axiom of otolaryngology that when the inner ear is involved in disease, cochlear and vestibular elements are compromised, which leads to hearing loss and vertigo. In cases of vestibular neuronitis, vertigo occurs in the absence of hearing loss, the inner ear is not involved, and the lesion lies in the vestibular neurons central to the labyrinth.

The absence of brainstem involvement suggests that the lesion is limited to the vestibular nerve. It is helpful to think of vestibular neuronitis as being a mononeuropathy, although this finding has not been confirmed. Whether the disease is caused by direct infection, localised thrombosis, or an autoimmune reaction remains unclear.

## 3. Infectious Hypothesis

An infective aetiology of vestibular neuronitis has long been hypothesised [29] on the basis of its association with respiratory tract and other infections and on its frequent occurrence in epidemics. Associations with preceding or concurrent infectious illnesses occur in 43% [14] to 46% of cases [3].

Several studies have demonstrated serological evidence of recent viral upper respiratory tract infections, particularly those caused by influenza virus A, influenza virus B, and adenoviruses, as well as infections caused by herpes simplex virus, cytomegalovirus, Epstein-Barr virus, rubella virus, and parainfluenza virus [30, 31]. Despite clear serological evidence of a recent viral infection, no virus has been isolated from the blood, respiratory tract, or cerebrospinal fluid of patients, despite repeat attempts [31].

**Figure 2:**
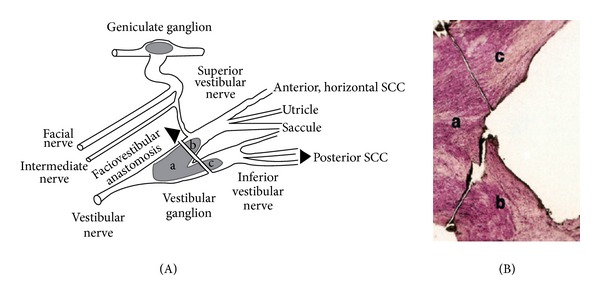
(A) schematic drawing of vestibular and facial nerves. Different section of vestibular ganglion (a) sterm, (b) inferior portion, and (c) superior portion. (B) Longitudinal section of human vestibular ganglion (a) sterm, (b) inferior portion, and (c) superior portion. Using polymerase chain reaction, herpes simplex virus 1 (HSV-1) DNA was found in 60% of the human esamine ganglia (from Arbusow et al.).

The histopathology of the vestibular nerve in cases of vestibular neuritis has exhibited atrophy of the vestibular nerve and the vestibular sensory epithelium. These results are similar to the histopathological findings in known viral disorders, such as herpes zoster oticus [26]. Herpes simplex virus type 1 (HSV-1) DNA has been detected at the time of autopsy with the use of the polymerase chain reaction in human vestibular ganglia [32] (Figure 2).

An animal model of vestibular neuritis was developed by inoculating HSV-1 into the auricle of mice [33]. In animals, vestibular symptomatology can also be induced by the intralabyrinthine inoculation of a variety of viral strains [34, 35]. In these types of experiments, the presence of viral antigens has been identified within the vestibular membranous labyrinth and Scarpa's ganglion cells [36]. Evidence for a viral infection in vestibular neuritis in humans is less convincing.

There are many similarities between VN and Bell palsy, and the reactivation of neurotropic viruses, including herpes simplex virus type 1, has been suggested as the cause of both conditions. Herpes simplex virus type 1 DNA is detected by polymerase chain reaction in approximately two-thirds of human vestibular and facial ganglia [32–37].

If HSV-1 is the most likely cause of VN, the virus would reside in a latent state in the vestibular ganglia. The virus could be located in the ganglionic nuclei, as has been reported in other cranial nerves [38, 39]. Other biological factors allow the virus to suddenly replicate, inducing inflammation and edema. This process causes secondary cell damage of the vestibular ganglion cells and axons in the bony canals.

Although the most popular theory of the pathogenesis of vestibular neuritis is based on a viral infection, the evidence to support this hypothesis remains circumstantial [40, 41].

## 4. Vascular Theory

Vestibular neuronitis is associated with an inflammatory event in acute episodes. Plasma fibrinogen and C-reactive protein (CRP) levels are increased, while lipoprotein (a) behaves as an acute-phase negative reactant [42].

There is increasing evidence for an inflammatory component that might be the result of a viral infection in the progression and aetiology of this disease [43, 44]. Patients treated with corticosteroids exhibited better clinical outcomes compared to patients treated with placebo. Virostatic drugs alone did not improve recovery [45, 46].

The CD40 receptor, which belongs to the tumour necrosis factor-*α* (TNF-*α*) family, and its ligand (CD40L) play important roles in the progression and genesis of inflammatory processes by the production of several proinflammatory cytokines, such as TNF-*α*, cellular adhesion molecules, or the expression of tissue factor [47].

TNF-*α* is a useful marker to monitor inflammatory processes, and it regulates the adhesion molecule CD38 [48] that mediates the transmigration of peripheral blood mononuclear cells (PBMCs) in interactions with CD31 on endothelial cells [49].

Kassner et al. [50] demonstrated an inflammatory activation of PBMCs in patients with VN and in patients with significantly elevated but not pathological levels of CRP compared to healthy persons. These findings support the hypothesis of a systemic reaction in these patients that is not only limited to the vestibular organ and supports the idea of a vascular component of the disease, which is similar to what is observed in sudden sensorineural hearing loss [51].

Freedman and Loscalzo found a significantly increased percentage of CD40-positive monocytes and macrophages in patients with VN compared to control subjects. An elevated expression of CD40 on monocytes/macrophages is known to cause the formation of platelet-monocyte aggregates, which contributes to thrombotic and inflammatory changes in the vascular system [52].

It is conceivable that these proinflammatory activated monocytes and macrophages might cause microvascular occlusion through the formation of platelet-monocyte aggregates by interactions with the CD40 receptor.

Several authors found a significantly positive expression of COX-2 in the B lymphocytes of patients with VN compared to healthy controls, which supports the idea of a proinflammatory state in patients with VN. COX-2 is a generally accepted inflammation marker and can be found in the PBMCs of patients with cardiovascular risk factors such as hyperlipidemia and smoking and in patients with other diseases, such as stroke and Alzheimer's dementia [53–56].

A proinflammatory activation of PBMCs is demonstrated by the significantly increased percentage of TNF-*α*-positive cells in the B lymphocyte and monocyte subpopulation of patients with VN. TNF-*α*, a generally accepted inflammation marker, activates leucocytes, enhances the adherence of neutrophils and monocytes to the endothelium, promotes the migration of inflammatory cells into the intercellular matrix, and triggers local production of other proinflammatory cytokines [57].

The results support the notion of a proinflammatory state in patients with VN. It can be hypothesised that the proinflammatory activation of PBMCs (and an elevation of CD40 in monocytes and macrophages) leads to reduced microvascular perfusion of the vestibular organ caused by an increase in thrombotic events. This hypothetical mechanism could cause a loss of function of the vestibular organ secondary to reduced perfusion and/or infarction.

Vascular occlusion as a cause of vestibular neuritis is not supportable on the basis of the peripheral vestibular histopathology.

## 5. Immunological Hypothesis

The characteristic interval that separates the onset of a respiratory tract infection and the onset of vertigo may suggest that the disease is caused by an immune mediated complication of the infection rather than direct viral infection of the nerve.

Immune mediated neurological disease is a well-recognised sequela of infectious fevers, and a parallel of localised immune mediated peripheral neuropathy is vaccine-induced brachial neuropathy that occasionally complicates deltoid immunisation.

Immunologic mechanisms have been suggested as possible causes for vestibular neuritis following influenza vaccination [58].

The view that vestibular neuronitis is immune mediated is supported by a study in which thymus lymphocytes-subpopulations (T4 T-helper and T8 T-suppressor cells) were found in inner ear diseases (e.g., sudden hearing loss, neuronitis vestibularis, Menière's disease, and Bell's Palsy) by specific monoclonal antibodies. The T-cell subset ratio (T4/T8) was elevated (less than 3) in approximately 50% of all patients. DR-typing was performed because of the well-known control of the immunoregulation through the class II HLA-DR antigens. There was a relative risk of 5.2 in peripheral vestibular lesion (neuronitis vestibularis). The relative risk of autoimmune diseases is found at this level [59].

Numerous and varied immunological tests have been developed in recent years for researching and diagnosing immunological diseases of the labyrinth. In spite of the different possibilities of immunological testing, Veldmann [60] is correct in the discussion of the diagnostic dilemma of supposed immunootological diseases like sudden hearing loss and Bell's palsy [61, 62].

This diagnosis involves immunoregulation, in which the T-helper lymphocytes (CD4)/T-suppressor lymphocyte CD8 ratio is determined. Diagnosis also involves a testing method to measure immunogenetics by HLA-DR typing. A simplified, hypothetical scheme for immunoregulation is that macrophages produce interleukin. This production causes the proliferation of CD4 helper cells, which leads to the activation of CD8 suppressor cells by interleukin [63].

B cells, which produce antibodies, are activated by CD4 helper cells and are impeded by CD8 suppressor cells. A pathological CD4/CD8 quotient appears in 57% of cases of Meniere's disease, 48% of cases of neuronitis vestibularis, and 39% cases of Bell's palsy. Sudden hearing loss is demonstrated as a pathological quotient in approximately 50% of patients [64].

There is only an imbalance in the CD4/CD8 quotient in inner ear diseases of unknown cause. This finding favours a causative immunological origin [65]. The primary question is whether the immunological imbalance CD4/CD8 is due to an increase in helper cells or a decrease in suppressor cells. The calculation of the absolute lymphocyte count demonstrates that the decrease of the total T lymphocytes is due to a suppression of both lymphocyte subpopulations. The evaluation indicates that the immunological imbalance is due to a greater percentage decrease of CD8 cells and not to an increase of CD4 helper cells.

The decrease of suppressor cells is an essential immunopathological finding. One hypothesis is that when the control of plasma is reduced through the decreased suppressor cells, the so-called “forbidden clones” become active. These “forbidden clones” may subsequently produce autoantibodies against the vestibular nerve. According to recent immunological knowledge, the immunological imbalance described above is most likely consistent with an autoimmune disease [64].

This immunological imbalance has a great similarity to findings in multiple sclerosis, as described by Reinherz et al. [66] and Bach et al. [63].

## 6. Treatment

The approach to VN patients has evolved since the 1980s with the introduction of early mobilisation, vestibular rehabilitation, and a reduced use of sedating vestibular suppressants [41].

The management of vestibular neuritis involves (1) symptomatic treatment with antivertiginous drugs to reduce vertigo, and nausea/vomiting, (2) causal treatment with corticosteroids to improve the recovery of peripheral vestibular function, and (3) physical therapy (vestibular exercises and balance training) to improve central vestibular compensation [67].

### 6.1. Symptomatic Treatment

It is generally believed that vestibular suppressants should be used only during the acute phase of the disease. This belief is based on the hypothesis that the protracted use of vestibular suppressants may impede central vestibular compensation.

The main classes of drugs used during the first 1 to 3 days for symptoms of acute vertigo include antihistamines, anticholinergic agents, antidopaminergic agents, and aminobutyric acid-enhancing (GABAergic) agents [41]. These drugs do not eliminate the vertiginous symptoms but reduce the severity of the symptoms.

The drugs are effective in most patients with vestibular neuritis, but there have been few controlled studies comparing the efficacy of these drugs [68]. There have been two recent randomised, clinical trials. One of these trials compared intravenous dimenhydrinate (50 mg) with lorazepam (2 mg) [69], and the other trial compared intramuscular dimenhydrinate (50 mg) with droperidol (2.5 mg) [70]. These trials determined that dimenhydrinate was more effective than lorazepam and that dimenhydrinate and droperidol were equally effective.

During the acute phase, severe nausea and decreased gastric motility make the intramuscular or intravenous route for drugs preferable. The response is clearly dose-dependent; therefore, if the initial dose is not effective, higher doses should be tried.

Although the exact mechanisms of action of these drugs are unclear, they act at the level of the neurotransmitters involved in the propagation of impulses from the primary to secondary vestibular neurons and in the maintenance of tone in the vestibular nuclei. The drugs also act on the areas of the nervous system that control vomiting, including the central components that are loosely described as the “emetic centre” and the peripheral components in the gastrointestinal tract.

All of the medications can be sedating; therefore, they should not be used when patients are engaged in activities that require a high level of alertness. Less sedating drugs, such as oral meclizine and transdermal scopolamine, are useful for milder cases of vertigo.

### 6.2. Causal Treatment

Monotherapy with steroids significantly improves the peripheral vestibular function of patients with vestibular neuritis. Treatment with methylprednisolone alone significantly improved the long-term outcomes of the peripheral vestibular function in patients with vestibular neuritis, while treatment with the antiviral agent (valacyclovir) did not improve the outcomes. The combination of these drugs was no more effective than methylprednisolone alone.

Methylprednisolone was administered daily as a single morning dose of 100 mg, and the dose was tapered every third day by 20 mg until the patient was receiving only 20 mg/day [46]. Valacyclovir was given as two 500 mg capsules three times daily for seven days.

Valacyclovir was used because the serum concentrations that result from its use are similar to those resulting from intravenous acyclovir [71] and because it is given at less frequent intervals than oral acyclovir.

Gianoli et al. [72] used 50 mg of prednisolone daily for 5 days and tapered the dose over the next 5 days. The effect of the lower doses does not seem to differ from that of the higher doses used by Strupp et al. [46].

The bony canal of the superior vestibular nerve is longer and narrower than the bony canal of the inferior vestibular nerve, and the superior vestibular nerve [73] might be more susceptible to entrapment from inflammatory swelling of the nerve. One of the glucocorticoid effects observed in both VN and Bell palsy might be decreased inflammation in the nerve with less oedema and less swelling of the nerve, which decreases the entrapment and reduces nerve damage.

Another important effect of steroids can also be depression of the immunological system. This finding supports the hypothesis that vestibular neuritis is an autoimmune disease similar to sudden hearing loss and Bell's palsy.

Animal experiments have shown that treatment with glucocorticoids accelerates vestibular compensation after unilateral vestibular lesions [74]. An improved central vestibular compensation can be another mechanism of glucocorticoid treatment of VN and might explain the quicker symptomatic improvement and shorter hospital stay.

### 6.3. Physical Therapy

Recovery from a peripheral vestibular lesion results from a combination of the restoration of peripheral labyrinthine function (which is usually incomplete in cases of vestibular neuritis) [75] and central vestibular compensation for the imbalance in vestibular tone.

Patients usually improve, even if they have a permanent unilateral loss of vestibular function. Clinicians have long felt that vestibular compensation occurs more rapidly and is more complete if the patient begins exercising as soon as possible after the occurrence of a vestibular lesion [76].

The goal of vestibular exercises is to accelerate the process of vestibular compensation and improve the final level of recovery. Vestibular exercises should be started when the acute stage of nausea and vomiting has ended.

A gradual program of physical exercise under the supervision of a physiotherapist improves the central vestibular compensation of a peripheral deficit. First, static stabilisation is focused on; then dynamic exercises are performed for balance control and gaze stabilisation during eye-head-body movements. The efficacy of physiotherapy in improving central vestibulospinal compensation in patients with vestibular neuritis has been demonstrated in a prospective, randomised, and controlled clinical study [77, 78].

## 7. Conclusions

Vestibular neuronitis is a disease in which vertigo occurs in the absence of hearing loss because the inner ear is not involved, and the lesion lies in the vestibular nerve. Vestibular neuritis is characterised by sudden and prolonged vertigo with the absence of auditory or neurological findings.

The most characteristic histopathology of vestibular neuritis in the human temporal bone is degeneration of the superior vestibular nerve and vestibular ganglion with variable involvement of the neuroepithelium of the end organs and a deficiency in the population of the nerve fibres and microscopic findings of myelin degeneration.

The vascular hypothesis is supported by the idea of a proinflammatory state and by the sudden onset of the disease. Further studies are needed to elucidate whether the interruption of the inflammatory changes in patients with VN can influence the recovery of the disease and the efficacy of clinical treatment.

Therapy with corticosteroids showed better clinical outcomes compared to placebo, while virostatics alone did not improve recovery. These results are in agreement with the hypothesis that VN is not a viral disease but is an autoimmune disease.

The most important issues requiring elucidation in the future include the pathogenesis of the disorder. The elucidation of the pathogenesis of vestibular neuritis may be facilitated by the use of molecular biological techniques on human temporal bone material and experimental animal models.

## 8. Take-Home Messages

- Vestibular neuronitis is a sudden unilateral loss of vestibular function and is the vestibular-nerve equivalent of sudden sensorineural hearing loss.
- In vestibular neuronitis, vertigo occurs in the absence of hearing loss; the inner ear is not involved, and the lesion is in the vestibular nerve. Vestibular neuronitis can be thought of as a mononeuropathy.
- The aetiology of the disease remains unclear. Vestibular neuronitis could be an autoimmune disease, but other pathological possibilities include viral infections and vascular disorders.
- The management of vestibular neuritis involves symptomatic treatment with antivertiginous drugs, causal treatment with corticosteroid, and physical therapy.
